# Supplementary material for: Content-rich biological network constructed by mining PubMed abstracts
Source: BMC Bioinformatics. 2004 Oct 8;5:147. doi: 10.1186/1471-2105-5-147 (PMC528731; doi:10.1186/1471-2105-5-147)
Supplement: Additional File 2 — The original results of the above study (non-essential files are deleted to keep the file size under the limit set by BMC bioinformatics). [file 1471-2105-5-147-S2.bz2 › chilibotAdditionalFile2/dip40/750ID9353297E18133/html/FHUA_TONB.html]

 


 **FHUA** and **TONB** 
  
Found 56 abstracts in PubMed, retrieved 40.  
 

 What does Google say? 
 PDF only 
| .edu only 

---

**Interactive relationship** (e.g. stimulation, inhibition, etc)

**Inhibitory relationship**- **TonB**  missense mutants reduced the activity of  **FhuA**  and FhuAdelta5 160.  Ref: 12427941 Microbiology, 2002
- In vivo synthesis of the periplasmic domain of  **TonB**  inhibits transport through the FecA and  **FhuA**  iron siderophore transporters of Escherichia coli.  Ref: 11566987 J Bacteriol, 2001
- Conversely, a monoclonal antibody that binds near the N terminus of  **FhuA**  reduced the retention of  **TonB**  by histidine tagged  **FhuA** .  Ref: 9353297 J Biol Chem, 1997
- In addition, the interactions that are inhibited by the  **TonB**  fragments must include more than the  **TonB**  box, since transport through corkless  **FhuA**  was also inhibited.  Ref: 11566987 J Bacteriol, 2001
- Overexpressed  **TonB**  33 239,  **TonB**  103 239, and  **TonB**  122 239 fragments inhibited transport of ferrichrome by  **FhuA**  and of ferric citrate by FecA, transcriptional induction of the fecABCDE transport genes by FecA, infection by phage phi80, and killing of cells by colicin M via  **FhuA** .  Ref: 11566987 J Bacteriol, 2001
- The activity of FhuADelta5 160 depended on  **TonB** , although the mutant lacks the  **TonB**  box residues 7 11 previously implicated in the interaction of  **FhuA**  with  **TonB** .  Ref: 10476037 Mol Microbiol, 1999
**Neutral relationship**- An in vitro system was established with which to examine the  **FhuA**   **TonB**  interaction.  Ref: 9353297 J Biol Chem, 1997
- Characterization of in vitro interactions between a truncated  **TonB**  protein from Escherichia coli and the outer membrane receptors  **FhuA**  and FepA.  Ref: 11292793 J Bacteriol, 2001
- Cross linking assays with the purified proteins in vitro demonstrated that there was preferential interaction between  **TonB**  and ligand loaded  **FhuA** .  Ref: 11292793 J Bacteriol, 2001
- **TonB**  of Escherichia coli activates  **FhuA**  through interaction with the beta barrel.  Ref: 12427941 Microbiology, 2002
- The transport active conformation of  **FhuA**  is mediated by a  **TonB**  induced conformational change in response to the energized cytoplasmic membrane.  Ref: 7688295 EMBO J, 1993
- It is proposed that  **TonB**  interacts with the  **TonB**  box of  **FhuA**  and with the beta barrel to release ferrichrome from the  **FhuA**  binding sites and to open the channel in  **FhuA** .  Ref: 10476037 Mol Microbiol, 1999
- Energy dependent receptor activities of Escherichia coli K 12 mutated  **TonB**  proteins alter  **FhuA**  receptor activities to phages T5, T1, phi 80 and to colicin M.  Ref: 8039674 FEMS Microbiol Lett, 1994
- These results demonstrate that the beta barrel domain of  **FhuA**  confers activity and specificity and responds to  **TonB** .  Ref: 11344156 J Bacteriol, 2001
- For the direct interaction of  **FhuA**  with  **TonB**  regions which are located outside the  **TonB**  box of  **FhuA**  and the region around residue 160 of  **TonB**  are important.  Ref: 8039674 FEMS Microbiol Lett, 1994
- Ligand binding to the ferrichrome iron receptor  [ **FHUA** ]   **fhua**  promotes interaction with the energy transducing protein  **TonB** .  Ref: 9353297 J Biol Chem, 1997
- It has been assumed that the proton motive force of the cytoplasmic membrane through the interaction of the  **TonB**  protein with the  **TonB**  box of the cork opens the  **FhuA**  channel.  Ref: 11344156 J Bacteriol, 2001
- these differences might contribute to the lower activity of the P. agglomerans  **TonB**  protein when coupled to the E. coli  **FhuA**  protein.  Ref: 12427941 Microbiology, 2002
- In vivo cross linking studies were performed to establish at the biochemical level the interaction between  **FhuA**  and  **TonB** .  Ref: 9353297 J Biol Chem, 1997
- In this paper we describe mutated  **FhuA**  proteins which displayed either an increased or decreased  **FhuA**  activity to phage T5 when combined with mutated  **TonB**  proteins.  Ref: 8039674 FEMS Microbiol Lett, 1994
- The hybrid  **FhuA**  proteins displayed a similar activity with the E. coli  **TonB**  protein as with their cognate  **TonB**  proteins.  Ref: 11344156 J Bacteriol, 2001
- FhuADelta5 160 still functions as a specific transporter, and sites in addition to the  **TonB**  box are involved in the  **TonB**  mediated response of  **FhuA**  to the proton gradient of the cytoplasmic membrane.  Ref: 10476037 Mol Microbiol, 1999
- The required energy is provided by the electrochemical potential of the cytoplasmic membrane which is assumed to induce a conformation of the  **TonB**  protein that causes a conformational change in  **FhuA**  so that bound ferrichrome is released into the periplasmic space located between the outer and the cytoplasmic membrane.  Ref: 7515827 FEBS Lett, 1994
- it was absent from strains of E. coli that were devoid of either  **FhuA**  or  **TonB** , and it was detected with anti  **FhuA**  and anti  **TonB**  monoclonal antibodies.  Ref: 9353297 J Biol Chem, 1997
- Yet, deletion of the cork results in an  **FhuA**  derivative, FhuADelta5 160, that still displays  **TonB**  dependent substrate transport and phage receptor activity.  Ref: 11344156 J Bacteriol, 2001
- It is proposed that the  **FhuA**  mutant proteins assume conformations which are either improved or impaired by the  **TonB**  derivatives.  Ref: 8039674 FEMS Microbiol Lett, 1994
- Excision of segments as small as 12 amino acids in the largest surface loop of  **FhuA**  converted  **FhuA**  into an open channel through which ferrichrome and antibiotics diffused independent of  **TonB**  ExbB ExbD.  Ref: 7515827 FEBS Lett, 1994
- By deleting 34 amino acid residues of a putative cell surface exposed loop,  **FhuA**  was converted from a ligand specific transport protein into a  **TonB**  independent and nonspecific diffusion channel.  Ref: 7688295 EMBO J, 1993
- Incubation of  **TonB**  with histidine tagged  **FhuA**  followed by addition of Ni2 nitrilotriacetate agarose led to the specific recovery of both  **TonB**  and  **FhuA** .  Ref: 9353297 J Biol Chem, 1997
- The data support the theory that the beta barrel receives the energy from the cytoplasmic membrane via  **TonB**  and responds to the energy input and thus represents the transporting domain of  **FhuA** .  Ref: 12427941 Microbiology, 2002
- The activity of the  **FhuA**  receptor in the outer membrane of Escherichia coli is dependent on the  **TonB** , ExbB and ExbD proteins which are anchored to the cytoplasmic membrane.  Ref: 8039674 FEMS Microbiol Lett, 1994
- However, deletion of the  **TonB**  box along with the globular domain results in a protein, FhuAdelta5 160, that still displays  **TonB**  dependent active ferrichrome transport across the outer membrane and confers sensitivity to the  **FhuA**  ligands.  Ref: 12427941 Microbiology, 2002
- The ferrichrome iron receptor  [ **FHUA** ]  of Escherichia coli is  **FhuA** , an outer membrane protein that is dependent upon the energy coupling protein  **TonB**  to enable active transport of specific hydroxamate siderophores, infection by certain phages, and cell killing by the protein antibiotics colicin M and microcin 25.  Ref: 9353297 J Biol Chem, 1997
- It is proposed that  **FhuA**  forms a closed channel which is opened by movement of the gating loop through a kind of allosteric interaction with  **TonB** .  Ref: 7515827 FEBS Lett, 1994
- **FhuA**  hybrids consisting of the beta barrel of one strain and the cork of another strain were active and showed higher  **TonB**  dependent ferrichrome transport rates than the corkless derivatives.  Ref: 11344156 J Bacteriol, 2001
- Each of the  **FhuA**  mutant proteins displayed activity for each of their ligands, except for phage T5, only when coupled to  **TonB** .  Ref: 11344156 J Bacteriol, 2001
- The energy source for active transport is the proton motive force of the cytoplasmic membrane, which is required for all  **FhuA**  functions except infection by phage T5, and is thought to be mediated to the outer membrane receptor  **FhuA**  by the  **TonB**  protein.  Ref: 12427941 Microbiology, 2002
- The 84kDa Irp protein exhibits features including a  **TonB**  box and a 50 amino acid region that can adopt occluded beta barrel structures similar to the plug domain of the Escherichia coli  **FhuA**  and FepA crystal structures.  Ref: 11524163 Vet Microbiol, 2001
- **FhuA** , the receptor for ferrichrome iron in Escherichia coli, is a member of a family of integral outer membrane proteins, which, together with the energy transducing protein  **TonB** , mediate the active transport of ferric siderophores across the outer membrane of Gram negative bacteria.  Ref: 9856937 Science, 1998
- Crystal structures of two outer membrane transport proteins,  **FhuA**  and FepA, and biochemical data reveal strong long range conformational changes of the proteins upon binding of Fe 3 siderophore complexes and in response to energy transfer from the cytoplasmic membrane into the outer membrane via the  **TonB**  ExbB ExbD protein complex.  Ref: 11934617 Curr Opin Microbiol, 2002
- Addition of ferricrocin or colicin M to  **FhuA**  in this system greatly increased the coupling between  **FhuA**  and  **TonB** .  Ref: 9353297 J Biol Chem, 1997
- These results indicate that, in vivo, the binding of ferricrocin to  **FhuA**  enhances complex formation between the receptor and  **TonB** .  Ref: 9353297 J Biol Chem, 1997

**Non-interactive relationship** (e.g. studied together, co-existance, homology, etc.)

- This inhibition by CN Cbl is increased when the expression of  **FhuA**  and  **TonB**  is repressed by growth with excess iron and is eliminated when BtuB synthesis is repressed by CN Cbl.  Ref: 7665457 J Bacteriol, 1995
- In addition, we found that transport across the outer membrane via  **FhuA** ,  **TonB** , ExbB, D rather than transport across the cytoplasmic membrane via FhuC, D, B was rate limiting.  Ref: 9613584 Mol Gen Genet, 1998
- Active transport of Fe3 as ferrichrome complex through the outer membrane of Escherichia coli is mediated by the  **FhuA**  outer membrane protein and the  **TonB**  ExbB ExbD protein complex in the cytoplasmic membrane.  Ref: 7515827 FEBS Lett, 1994
- Most of the  **FhuA**  derivatives which were not affected by Llp were, according to a previously published  **FhuA**  transmembrane topology model, located in periplasmic turns and in the  **TonB**  box close to the periplasm.  Ref: 8045901 J Bacteriol, 1994
- Phenotypic and mapping studies showed the mutations to be located in the  **fhuA** , exb,  **tonB** , and sbmA genes.  Ref: 7768835 J Bacteriol, 1995
- We propose a plausible model of binding of  **TonB**  to  **FhuA**  and FepA, two  **TonB**  dependent outer membrane receptors.  Ref: 11328822 J Biol Chem, 2001
- Ferrichrome iron is actively transported across the outer membrane of Escherichia coli by the  **TonB**  dependent receptor  **FhuA** .  Ref: 8939430 Mol Microbiol, 1996
- It is proposed that by such an alteration of  **FhuA**  conformation,  **TonB**  is triggered to energize the active transport of the bound ligand across the outer membrane.  Ref: 8939430 Mol Microbiol, 1996
- DALTV of FecA could be functionally replaced with the core  **TonB**  boxes of  **FhuA**  DTITV and FepA DTIVV.  Ref: 12618451 J Bacteriol, 2003
- **TonB**  proteins of different Enterobacteriaceae activated  **FhuA**  and FhuAdelta5 160 to a similar degree.  Ref: 12427941 Microbiology, 2002
- Although these receptors may vary in complexity and composition, the key protein involved in the transport of iron as iron, haem or iron siderophore across the outer membrane is a  **TonB**  dependent receptor with an overall structure presumably similar to that determined recently for Escherichia coli  **FhuA**  or FepA.  Ref: 10383753 Mol Microbiol, 1999
- FyuA shows homology to other iron regulated  **TonB**  dependent outer membrane proteins with receptor functions e.g. BtuB, CirA, FepA, IutA,  **FhuA** , FoxA, FcuA.  Ref: 7984105 Mol Microbiol, 1994
- The crystallographic structures of two  **TonB**  dependent receptors  **FhuA**  and FepA have recently been determined.  Ref: 12409204 Biochim Biophys Acta, 2002
- H 6  **TonB**  captured both the ferrichrome iron receptor  [ **FHUA** ]   **FhuA**  and the ferric enterobactin receptor FepA from detergent solubilized outer membranes in vitro.  Ref: 11292793 J Bacteriol, 2001
- We consider a variety of different colicins, including A, B, D, E1, E3, Ia, M and N, that penetrate through the porins OmpF, FepA, BtuB, Cir and  **FhuA** , to subsequently interact with a few targets in the periplasm, including TolA, TolB, TolC and  **TonB** .  Ref: 12423783 Biochimie< MedlineTA>Biochimie, 2001
- In this study synthetic nonapeptides identical in sequence to amino acids 150 15 51 15 52 16 53 161 and 158 166 of  **TonB**  were shown to reduce ferrichrome transport of cells via wild type  **FhuA**  and the corkless derivative FhuAdelta5 160, which suggests that this  **TonB**  region is involved in the interaction of  **TonB**  with the beta barrel of  **FhuA** .  Ref: 12427941 Microbiology, 2002
- The crystallographic structures of the  **TonB**  dependent transporter  **FhuA**  revealed that the region near the Ton box, which itself was not resolved, is exposed to the periplasmic space and undergoes an extensive shift in position upon binding of substrate.  Ref: 11029413 J Bacteriol, 2000
- They include the OmpA membrane domain, the OmpX protein, phospholipase A, general porins OmpF, PhoE, substrate specific porins LamB, ScrY and the  **TonB**  dependent iron siderophore transporters  **FhuA**  and FepA.  Ref: 10931321 Mol Microbiol, 2000
- Unwinding of the switch helix is proposed to be required for efficient binding of  **TonB**  to  **FhuA**  and for coupling the proton motive force of the cytoplasmic membrane with energy dependent ligand transport.  Ref: 11587645 Structure Camb , 2001
- CONCLUSIONS We conclude that the binding of rifamycin CGP 4832 destabilizes the switch helix and promotes the formation of a transport competent  **FhuA**   **TonB**  complex, albeit with lower efficiency than ferrichrome.  Ref: 11587645 Structure Camb , 2001
- The beta barrel domain of FhuADelta5 160 is sufficient for  **TonB**  dependent  **FhuA**  activities of Escherichia coli.  Ref: 10476037 Mol Microbiol, 1999
- These results suggest conformational changes in  **FhuA**  by  **TonB**  which are recognized by phage T5.  Ref: 8039674 FEMS Microbiol Lett, 1994
- However, growth of a FhuADelta5 160  **tonB**  wild type strain occurred at low ferrichrome concentrations, and ferrichrome was transported at about 45% of the  **FhuA**  wild type rate despite the lack of ferrichrome binding sites provided by the cork.  Ref: 10476037 Mol Microbiol, 1999
- The transport is coupled to the proton motive force, which energizes  **FhuA**  through the inner membrane protein  **TonB** .  Ref: 11587645 Structure Camb , 2001
- Complementation studies indicated that the neisserial Ton system cannot interact with the E. coli  **FhuA**   **TonB**  dependent outer membrane receptor.  Ref: 9006036 J Bacteriol, 1997
